# Genomic insights into the evolution of hybrid isoprenoid biosynthetic gene clusters in the MAR4 marine streptomycete clade

**DOI:** 10.1186/s12864-015-2110-3

**Published:** 2015-11-17

**Authors:** Kelley A. Gallagher, Paul R. Jensen

**Affiliations:** Center for Marine Biotechnology and Biomedicine, Scripps Institution of Oceanography, University of California San Diego, 9500 Gilman Drive, La Jolla, CA 92093-0204 USA

**Keywords:** Biosynthetic gene clusters, Genome evolution, *Streptomyces*, ABBA prenyltransferases, Secondary metabolism, Hybrid isoprenoids

## Abstract

**Background:**

Considerable advances have been made in our understanding of the molecular genetics of secondary metabolite biosynthesis. Coupled with increased access to genome sequence data, new insight can be gained into the diversity and distributions of secondary metabolite biosynthetic gene clusters and the evolutionary processes that generate them. Here we examine the distribution of gene clusters predicted to encode the biosynthesis of a structurally diverse class of molecules called hybrid isoprenoids (HIs) in the genus *Streptomyces*. These compounds are derived from a mixed biosynthetic origin that is characterized by the incorporation of a terpene moiety onto a variety of chemical scaffolds and include many potent antibiotic and cytotoxic agents.

**Results:**

One hundred and twenty *Streptomyces* genomes were searched for HI biosynthetic gene clusters using ABBA prenyltransferases (PTases) as queries. These enzymes are responsible for a key step in HI biosynthesis. The strains included 12 that belong to the ‘MAR4’ clade, a largely marine-derived lineage linked to the production of diverse HI secondary metabolites. We found ABBA PTase homologs in all of the MAR4 genomes, which averaged five copies per strain, compared with 21 % of the non-MAR4 genomes, which averaged one copy per strain. Phylogenetic analyses suggest that MAR4 PTase diversity has arisen by a combination of horizontal gene transfer and gene duplication. Furthermore, there is evidence that HI gene cluster diversity is generated by the horizontal exchange of orthologous PTases among clusters. Many putative HI gene clusters have not been linked to their secondary metabolic products, suggesting that MAR4 strains will yield additional new compounds in this structure class. Finally, we confirm that the mevalonate pathway is not always present in genomes that contain HI gene clusters and thus is not a reliable query for identifying strains with the potential to produce HI secondary metabolites.

**Conclusions:**

We found that marine-derived MAR4 streptomycetes possess a relatively high genetic potential for HI biosynthesis. The combination of horizontal gene transfer, duplication, and rearrangement indicate that complex evolutionary processes account for the high level of HI gene cluster diversity in these bacteria, the products of which may provide a yet to be defined adaptation to the marine environment.

**Electronic supplementary material:**

The online version of this article (doi:10.1186/s12864-015-2110-3) contains supplementary material, which is available to authorized users.

## Background

In bacteria, the genes responsible for the biosynthesis of secondary metabolites are typically clustered on the chromosome [1]. These biosynthetic gene clusters evolve rapidly relative to other genetic elements [2, 3], which likely contributes to the remarkable structural diversity observed among secondary metabolites. While the selective pressures driving secondary metabolite diversification remain largely unknown, the availability of large numbers of genome sequences has made it possible to begin to identify the evolutionary mechanisms that govern the biogenesis of structural novelty [2–4].

The genus *Streptomyces* is well known as the source of structurally diverse secondary metabolites [5, 6]. With nearly 600 named species, it is the most specious of all bacterial genera and comprises the majority of diversity within the family Streptomycetaceae. Streptomycetes are typically saprophytic and found in terrestrial soils and marine sediments. They also occur as plant endophytes, invertebrate mutualists, and human or plant pathogens [7, 8]. Within the genus *Streptomyces*, the ‘MAR4’ clade has been described as a largely marine-derived lineage [9]. Based on available sequence data, it currently encompasses 57 cultured strains and 180 cloned sequences and displays 4.1 % 16S rRNA divergence. Members of this clade consistently form two sub-clades represented by the type strains *S. aculeolatus* and *S. synnematoformans* [9], which are the only named species within the MAR4 lineage.

MAR4 strains have previously been linked to the production of secondary metabolites broadly classified as hybrid isoprenoids (HIs) [10]. These compounds are biosynthetic hybrids that derive part of their structures from five-carbon isoprene units. The addition of isoprene (a process called “prenylation”) can occur on a variety of chemical scaffolds thus creating considerable structural diversity. HIs frequently possess biological activity and thus their discovery is of interest to the pharmaceutical industry [11]. Based on literature reports, HI production appears to be scattered throughout the *Streptomyces* phylogeny and is a relatively rare part of secondary metabolism [10]. In contrast, some members of the MAR4 clade have been observed to produce up to three distinct classes of HI secondary metabolites [10, 12, 13]. In addition, all MAR4 strains tested produce at least one HI, an ability that has not been reported elsewhere in the *Streptomyces* genus [9]. To date, HI secondary metabolites produced by MAR4 strains include naphthoquionones in the napyradiomycin [14] and marinone class [15, 16], the phthalazinone azamerone [17], the phenazines lavanducyanin [18] and marinophenazine [13], and the highly unusual pyrrole nitropyrrolin [12].

Prenyltransferases (PTases) are responsible for the attachment of isoprene moieties to a variety of acceptor molecules and thus play a critical role in the biosynthesis of HI secondary metabolites. These enzymes also play important roles in the biosynthesis of primary metabolites including membrane sterols and lipoquinones [19]. One sub-group of PTases specific to secondary metabolism are the ABBA PTases, named in reference to the ααββ structural repeats that form a large β-barrel fold comprising the active center of the enzyme. ABBA PTases attach isoprene moieties to aromatic substrates and, to date, all characterized ABBA PTases are involved in the biosynthesis of HI secondary metabolites [20].

ABBA PTases can be further divided into two sub-groups, both of which are found in fungi and bacteria. The first group is the indole ABBA PTases, which are responsible for the prenylation of indole substrates. One example is the enzyme CymD, which is responsible for prenylation of the bacterial cyclic peptide cyclomarin [21]. The second group is the ‘Orf2’ PTases, which attach isoprenoid moieties to a variety of aromatic substrates including phenazines, naphthoquinones, and aminocoumarins. This group is named for the first characterized member, later renamed ‘NphB’, which is involved in naphterpin biosynthesis [22]. Although the indole and Orf2 PTases bear little sequence homology, they are thought to have arisen from a common ancestor [20]. To the best of our knowledge, the unrelated PTase CnqPT1 is the only non-ABBA PTase involved in the production of a HI secondary metabolite. CnqPT1 is membrane-bound and responsible for the O-prenylation of phenazine in the biosynthesis of marinophenazine in the MAR4 strain CNQ-509 [13].

The isoprene used in bacterial terpenoid biosynthesis is derived from either the mevalonate (*mev*) or non-mevalonate pathways [23]. Most bacteria, including *Streptomyces*, possess the non-mevalonate pathway, the products of which are used in the biosynthesis of primary metabolites such as respiratory ubiquinones [24, 25]. In cases where bacteria also possess the *mev* pathway in addition to the non-mevalonate pathway, it has always been found flanking a HI gene cluster [26] suggesting that the isoprene produced by this biosynthetic route is used for secondary metabolism. Feeding experiments have confirmed that the *mev* pathway can provide some or all of the isoprene incorporated into HI secondary metabolites [23, 27–30] and led to the hypothesis that the *mev* pathway is a marker for HI production [26]. The availability of a large number of *Streptomyces* genome sequences now provides the opportunity to further explore the association of the *mev* pathway with HI biosynthesis.

To date, most evidence for the presence of HI pathways in MAR4 strains is based the detection and/or isolation of HI secondary metabolites from MAR4 strains [9, 12, 13, 15, 16, 31, 32]. Here we use comparative genomics to more rigorously test the hypothesis that MAR4 strains are enriched in HI gene clusters relative to other streptomycetes. We show that MAR4 PTases have undergone gene duplication events and been exchanged among unrelated gene clusters. We also show that most strains possessing HI gene clusters do not contain the *mev* pathway, suggesting it is not a good marker for the presence of this type of biosynthetic capacity. The evolutionary history of HI pathways in MAR4 streptomycetes provides insight into how chemical diversity is generated in microbial secondary metabolism.

## Results

### ABBA prenyltransferases in *Streptomyces* genomes

**Fig. 1:**
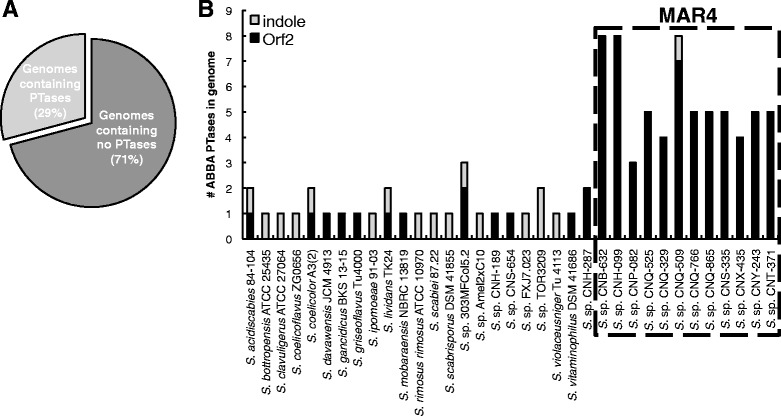
PTase distributions among genome sequences. **a** Percentage of genomes that contain ABBA PTases. **b** Distribution of indole and Orf2 ABBA PTases among the 35 strains that possessed these genes

One hundred and twenty *Streptomyces* genome sequences were analyzed including 11 that were acquired from MAR4 strains as part of this study (Additional file 1: Table S1). These genomes were initially screened for the presence of HI biosynthetic gene clusters (HIBGCs) using 17 experimentally characterized Orf2 and indole ABBA PTases as BLAST search queries (Additional file 1: Table S2). The top BLAST matches were incorporated into a phylogeny that included experimentally characterized enzymes representing both PTase sub-classes [20] (data not shown). In total, 95 PTases claded with either the Orf2 or indole ABBA PTase lineages (Additional file 1: Table S3). These sequences showed varying levels of identity to the queries (29–100 % AA sequence identity), yet all were assigned to ABBA PTase-specific pfam families. The 95 ABBA PTases were distributed among 35 strains (29.2 % of the total), some of which contained more than one of these genes (Fig. 1). The 12 MAR4 strains contained from 3 to 8 ABBA PTases per genome. With the exception of a single indole PTase in strain CNQ-509, all of these fell within the Orf2 class. Of the 108 non-MAR4 strains, 23 contained ABBA PTases. Of these, one contained three, five contained two, and 17 contained one copy of this gene. In contrast to the MAR4 ABBA PTases, these could be delineated into 16 indole and 14 Orf2 PTases.

### Distribution of ABBA PTases among *Streptomyces* spp

**Fig. 2:**
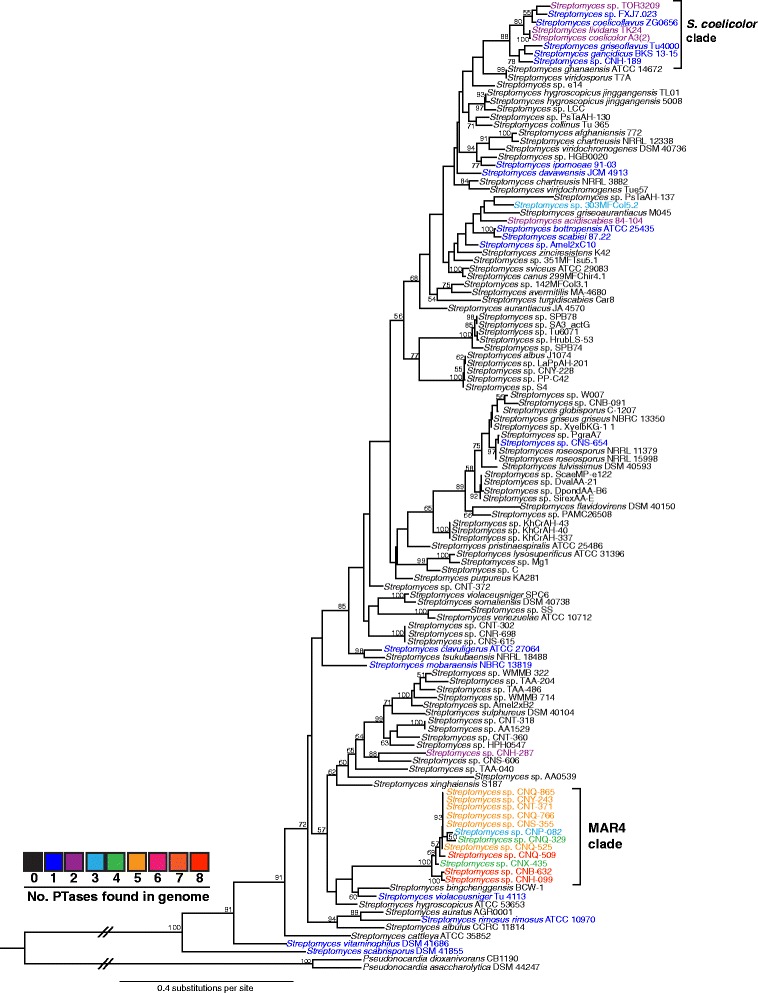
Maximum likelihood phylogeny of the 120 *Streptomyces* strains used in this study. Phylogeny is based on concatenated AtpD and RpoB amino acid sequences. Bootstrap values >50 % are indicated at their respective nodes (based on 100 replicates). Colors indicate the number of ABBA PTases found in each genome. The MAR4 and *S. coelicolor* clades are indicated. Sequences derived from two *Pseudonocardia* genomes were used to root the tree

The phylogenetic relationships of the 35 strains that contained ABBA PTases were examined in the context of a *Streptomyces* species tree that included the 120 strains used in this study (Fig. 2). This phylogeny was generated from the shared, single copy housekeeping genes AtpD and RpoB. The MAR4 clade is well delineated within this phylogeny, which supports previous 16S rRNA gene sequence analyses [9]. The results clearly show that ABBA PTases are sparsely distributed throughout the genus with the exception of the MAR4 clade and a distantly related clade designated ‘*S. coelicolor*’ in reference to its best-characterized member. These two clades are composed entirely of ABBA PTase-containing strains, with members of the MAR4 clade containing on average five ABBA PTases per genome compared with one per genome for members of the *S. coelicolor* clade. The sporadic distribution of ABBA PTases throughout the tree largely conforms to previous reports of HI production in this genus [10].

### Orf2 ABBA PTase phylogeny

**Fig. 3:**
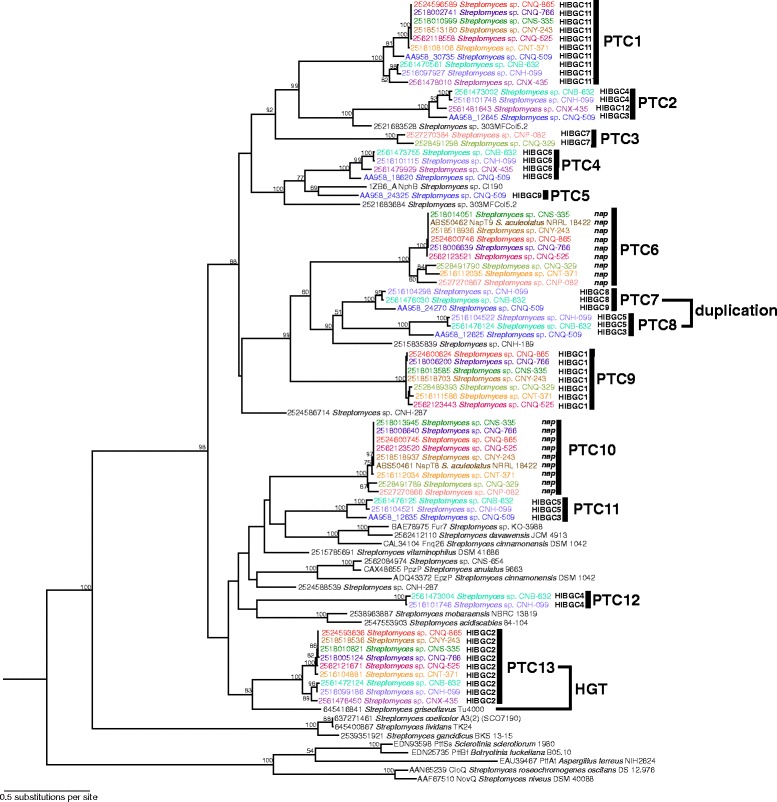
Maximum likelihood phylogeny of Orf2 ABBA PTases with mid-point rooting. The phylogeny contains all of the Orf2 ABBA PTases identified in the 120 *Streptomyces* genomes (identified by IMG gene number) including 12 that are experimentally characterized (identified by accession number). Homologs found in non-MAR4 genomes are in black, while those from MAR4 strains are color-coded based on strain. MAR4 PTases are delineated into 13 prenyltransferase clades (PTCs). Each MAR4 PTase is also assigned a hybrid isoprenoid gene cluster (HIBGC) number, which defines the gene cluster in which it was observed. Bootstrap values >50 % are indicated at their respective nodes (based on 100 replicates). Examples of horizontal gene transfer (HGT) and gene duplication are indicated. Two of the characterized PTases (ABS50462 and ABS50461) are from the MAR4 strain *S. aculeolatus* NRRL 18422

An Orf2 ABBA PTase phylogeny was constructed to assess the evolutionary relationships among the sequences identified in this study (Fig. 3). The initial bifurcation in the Orf2 phylogeny reveals two clades, the smaller of which appears at the bottom of the tree and contains the previously characterized CloQ and NovQ PTases, which are responsible for the prenylation of the aminocoumarin molecules chlorobiocin and novobiocin, respectively [33]. None of the MAR4 sequences fall in this clade. The larger, sister clade includes PTases known to prenylate naphthoquinone (e.g. NphB, Fur7, and Fnq26) and phenazine (EpzP and PpzP) scaffolds [20]. All of the PTases found in the MAR4 genomes fell within this larger clade. The MAR4 PTases could be further delineated into thirteen highly supported sub-clades. These sub-clades were each assigned prenyltransferase clade (PTC) numbers (Fig. 3). PTC5 contains a single prenyltransferase but was sufficiently separated from the other sequences to assign it an independent clade number. The only two experimentally characterized Orf2 PTases that clade with MAR4 sequences are both from the napyradiomycin (*nap*) biosynthetic pathway (ABS50462 and ABS50461) [14].

### Orf2 evolution

By comparing the Orf2 phylogeny (Fig. 3) with the species tree (Fig. 2), it becomes clear that closely related PTases occur in distantly related strains. For example, the PTase that is sister to PTC13 occurs in *S. griseoflavus* Tu4000, which is distantly related to the MAR4 clade in the *Streptomyces* phylogeny. This suggests that these PTases have been exchanged by HGT. In other cases, there is evidence of PTase duplication followed by divergence. This is demonstrated for the sister PTC7 and PTC8 clades, which share the same phylogenies and occur in the same taxa (Fig. 3).

**Fig. 4:**
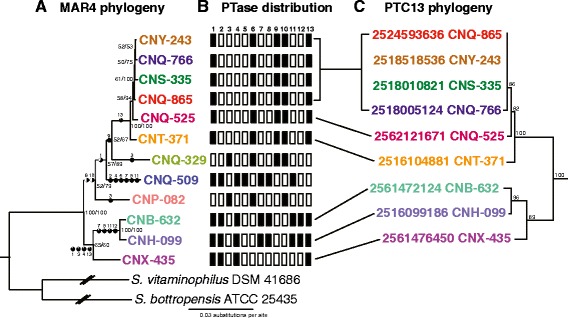
Evolutionary relationships between MAR4 strains and PTases. **a** MAR4 phylogeny generated from five single-copy housekeeping genes (*atpD*, *rpoB*, *trpB*, *recA*, and *gyrB*). Maximum likelihood (ML) and maximum parsimony (MP) trees showed the same topology. Bootstrap values >50 % are indicated at the respective nodes (MP/ML) based on 100 replicates. Black circles and associated PTC numbers indicate predicted PTase acquisition points with the % fill of the circle indicating the proportional likelihood that the PTase was present at that node (only values ≥50 % are shown). Note: some PTases are predicted to have been acquired at multiple nodes. **b** Boxes depict PTase distributions among MAR4 strains (black = present, white = absent). Numbers indicate the PTC. **c** The PTase phylogeny for each PTC was largely congruent with that of the species phylogeny for the strains in which it was observed. As an example, the phylogeny of PTC13 is depicted in comparison with the strain phylogeny. The likelihood analysis predicts two independent acquisition events for PTC13 after which the PTase phylogeny supports a model of vertical inheritance. Bootstrap values >50 % are indicated at the respective nodes (ML). IMG gene ID and the source strain for each PTase are given

To better resolve the relationships among the MAR4 strains and the Orf2 PTases, a species phylogeny was generated using five single-copy housekeeping genes derived from the 12 MAR4 genome sequences (Fig. 4). A likelihood analysis was then used to predict the ancestral node for each PTase based on their distributions within the phylogeny. This analysis predicts that no PTases were present in the MAR4 common ancestor, suggesting that all were acquired by HGT. In addition, it is predicted that nine of 13 PTases were acquired more than once within the MAR4 clade while three were lost in certain strains following acquisition events. Specifically, PTC6 and PTC10 are predicted to have been lost in the CNQ-509 lineage, while PTC1 is predicted to have been lost in the CNQ-329 lineage. Five PTases are found in seven or more MAR4 strains (PTCs 1,6,9,10,13) while all others (2–5, 7–8, and 11–12) are present in four or fewer. In all cases, the phylogeny within each PTC (Fig. 3) is largely congruent with that of the MAR4 species phylogeny (Fig. 4) suggesting that, once acquired, these genes follow a vertical model of inheritance.

### Hybrid isoprenoid biosynthetic gene clusters (HIBGCs)

**Fig. 5:**
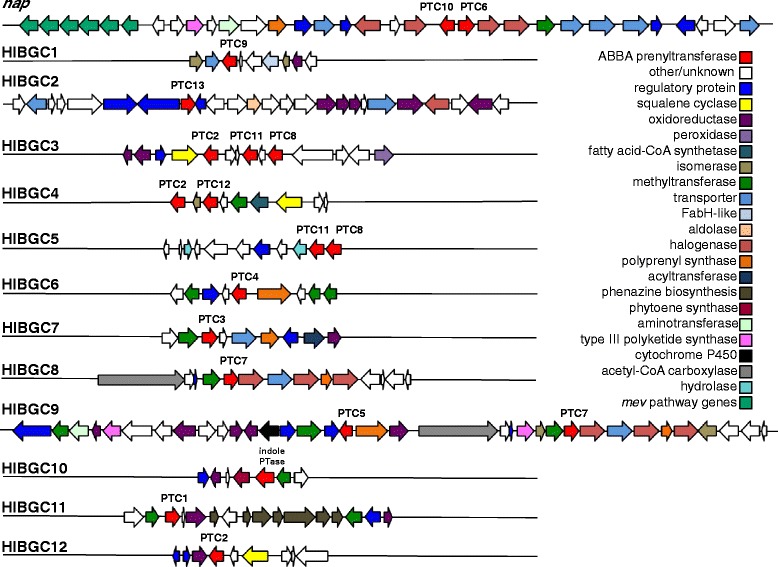
Representative putative HI biosynthetic gene clusters (HIBGCs) identified in MAR4 strains. Putative gene functions (predicted by pfam annotations) are indicated by color. The PTC associated with each PTase is indicated

We next analyzed the gene neighborhoods surrounding each MAR4 PTase and sought to identify gene clusters that putatively encode for the biosynthesis of HI secondary metabolites. The HI biosynthetic gene clusters (HIBGCs) observed in different strains were then grouped when shared gene content and MultiGeneBlast [34] analyses revealed sufficient similarity to predict they encode structurally related secondary metabolites. This led to the identification of 13 HIBGCs of which only one could be linked to a previously characterized pathway (*nap*, Table 1, Fig. 5) [14, 28]. It is generally difficult to predict the scaffold that will be prenylated based on HIBGC gene content or Orf2 phylogeny and thus little can be inferred about the secondary metabolites encoded by the remaining 12 gene clusters. Nonetheless, HIBGC9 contains a type III polyketide synthase, which would be expected for the production of the naphthoquinone moiety of compounds in the marinone series (Fig. 5). Likewise, HIBGC11 contains a full suite of phenazine biosynthesis genes and is therefore predicted to encode the production of the known MAR4 HI lavanducyanin. However, these bioinformatic predictions remain to be experimentally verified.

**Table 1:** Summary of putative HI gene clusters (HIBGCs) identified in MAR4 strains. The PTases present in each HIBGC are indicated along with the annotation of key biosynthetic genes

Gene cluster	PTC(s) present	HIBGC content	Product
*nap*	PTC10, PTC6	Type III polyketide synthase, three haloperoxidases, *mev* pathway	napyradiomycin
HIBGC1	PTC9	FabH-like protein, AvrD-like protein	Unknown
HIBGC2	PTC13	haloperoxidase	Unknown
HIBGC3	PTC2, PTC8, PTC11	squalene-hopene cyclase	Unknown
HIBGC4	PTC2, PTC12	squalene-hopene cyclase	Unknown
HIBGC5	PTC8, PTC11	Found on very short contig, additional genes in this cluster may exist	Unknown
HIBGC6	PTC4	isopentenyl pyrophosphate synthase	Unknown
HIBGC7	PTC3	polyprenyl synthase	Unknown
HIBGC8	PTC7	three haloperoxidases; found on very short contig, additional genes in this cluster may exist	Unknown
HIBGC9	PTC7, PTC5	Two type III polyketide synthases; portion of this HIBGC is identical to HIBGC8	marinone^a^
HIBGC10	N/A	indole PTase, phytoene synthase	Unknown
HIBGC11	PTC1	full suite of phenazine biosynthesis genes (*phzABCDEFG*)	lavanducyanin^a^
HIBGC12	PTC2	squalene-hopene cyclase	Unknown

^a^Predicted

Two HIBGCs (8 and 9) did not meet the criteria to be assigned to the same cluster, however their gene content is largely identical except that HIBGC9 contains an additional set of genes including a second PTase and a type III polyketide synthase. Because HIBGC8 occurs on a relatively short contig, further sequencing is required to determine if these two clusters are in fact the same. Four of the HIBGCs contain more than one PTase (Figs. 3, 5, Table 1). In each of these cases, the PTases are distantly related to each other (i.e. they occur in different PTCs). For example, HIBGC4 contains two PTases that belong to PTCs 2 and 12 (Fig. 3) and thus appear to have been independently recruited into the cluster. This is supported by the likelihood analysis, which predicts that PTC2 and PTC12 were acquired at different points in the evolutionary history of strains CNB-632 and CNH-099 (Fig. 4). Overall, the varying gene content among the MAR4 HIBGCs reveals the potential biosynthetic diversity maintained by these strains.

### Evidence for PTase rearrangement

**Fig. 6:**
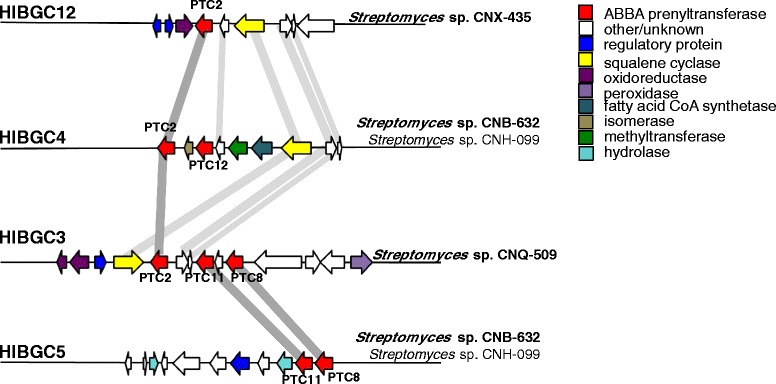
PTase rearrangement within MAR4 strains. Four HIBGCs are illustrated with putative gene functions (predicted by pfam annotations) indicated by color. Homologs are indicated by gray bars. ABBA PTases are shown in red, and the PTC associated with each PTase indicated. The strain(s) containing each HIBGC are labeled

In most cases, all of the PTases that fall within a single PTC occur in the same HIBGC (Fig. 3). There are, however, three notable exceptions (PTCs 2, 8, and 11), where orthologous PTases are found in different HIBGCs. A closer look reveals that the four PTC2 sequences are found in three different HIBGCs, each of which contains from 1 to 3 PTases (Fig. 6). The presence of orthologous PTases in different gene clusters suggests they are actively exchanged and can be involved in the production of distinct secondary metabolites. Thus, PTase phylogeny is not a good predictor of the secondary metabolites produced by HIBGCs.

### Mevalonate pathway distribution

The 120 *Streptomyces* genomes were examined for the six genes that comprise the *mev* pathway to determine its association with HIBGCs. Thirteen genomes were found to contain the complete *mev* pathway (Additional file 1: Table S4). Of these, seven were MAR4 strains. In contrast, 35 strains contained ABBA PTases. The *mev* pathway was never found more than once in a single genome. Of the 13 strains that contained the *mev* pathway, 11 contained at least one ABBA PTase. In the two strains that contained the *mev* pathway but lacked an ABBA PTase (*Streptomyces* sp. TAA-040 and *Streptomyces* sp. CNT-372), the *mev* pathway was found in close proximity to genes involved in terpene biosynthesis (i.e. a terpene synthase or non-ABBA PTases) suggesting it may be involved in the biosynthesis of terpenoid secondary metabolites.

The seven MAR4 strains that contained the *mev* pathway also contained the *nap* gene cluster, supporting previous reports that the isoprene incorporated into the napyradiomycins is derived from *mev* [28, 30]. However, the *mev* pathway was often found on short contigs making it difficult to establish its proximity to the *nap* pathway. In all other cases, the *mev* pathway was found in close proximity to an ABBA PTase (Additional file 1: Table S4). Thus, it appears that when present, the *mev* pathway is generally associated with genes for secondary metabolism, however these genes do not appear to be consistently associated with HIBGCs and are often absent in strains with the genetic potential to produce HIs.

## Discussion

Access to genome sequence data is providing unprecedented opportunities to explore the evolution of secondary metabolite biosynthesis [3, 4, 35]. Studies in this area have largely focused on modular enzyme systems, such as those encoding non-ribosomal peptides and polyketides. In contrast, relatively little is known about the diversity and distributions of gene clusters responsible for HI biosynthesis and how these systems evolve to create new secondary metabolite diversity. HIs encompass a wide spectrum of biosynthetic paradigms, however a consistent feature of the associated gene clusters is the presence of PTases, which facilitate the addition of isoprene moieties to the secondary metabolite.

Using a dataset of 120 *Streptomyces* genomes, we provide additional bioinformatic support for the hypothesis that MAR4 streptomycetes are enriched in PTases relative to other streptomycetes [10]. Interestingly, all but one of the MAR4 PTases falls within the Orf2 class of ABBA PTases. This is unlike non-MAR4 streptomycetes, which contain approximately equal numbers of indole and Orf2 PTases. ABBA PTases were also enriched in the *S. coelicolor* A3(2) clade, however these strains contained on average one copy per genome compared to five in MAR4 strains. Outside of these two clades, PTases were sparsely distributed throughout the *Streptomyces* species tree.

Likelihood analyses predict that the MAR4 PTases were acquired via multiple HGT events (Fig. 4). The alternative and less parsimonious hypothesis is that the PTases were acquired via HGT by a common MAR4 ancestor and subsequently lost by many of the strains. Regardless of which scenario is correct, HGT appears to have played a major role in the acquisition of PTases by MAR4 strains (Fig. 2). There is also evidence that PTases have been lost in some lineages, duplicated in others, and exchanged among HIBGCs, revealing the dynamic evolutionary processes that are acting on this enzyme class. The consistent occurrence and relatively high abundance of PTases in MAR4 strains suggests they provide a selective advantage to these bacteria.

All of the MAR4 PTases could be assigned to 13 HIBGCs, each of which is predicted to encode the production of a distinct HI molecule. While there was generally a strong correlation between the phylogenetic relationships among the PTases and the HIBGCs in which they occurred, there was also clear evidence that PTases are exchanged among HIBGCs (e.g., PTases in PTC2 were observed in three different HIBGCs, Fig. 6). While it might be assumed that PTase duplication followed by divergence would be a primary mechanism by which HIBGCs evolve to generate new structural diversity, there were no cases where a single HIBGC contained paralogous PTases. In fact, in all cases where multiple PTases were found in the same gene cluster (HIBGCs 3, 4, 5, and 9) the PTases were distantly related to each other (Fig. 3), and in one case (HIBGC4), PTases in the same cluster are predicted to have been acquired at different points during MAR4 evolution (Fig. 3). ABBA PTases have been shown to prenylate a variety of substrates in vitro [20], suggesting they could be functionally incorporated into new HIBGCs. These results suggest that a primary mechanism of HIBGC evolution is the acquisition of new PTases. In the one case where there is clear evidence for PTC duplication (PTC 7 and 8), the paralogous PTases in each stain occur in different HIBGCs. Given that some orthologous PTases occurred in different HIBGCs, which implies they encode the biosynthesis of distinct molecules (Fig. 6), a generalized conclusion of this study is that PTase phylogeny is not a reliable predictor of HIBGC content and, by extension, secondary metabolite production. Furthermore, PTase exchange among gene clusters appears to be a mechanism by which novel HIBGCs evolve. This finding is in accordance with a recent study of biosynthetic gene cluster evolution which showed that certain portions of gene clusters can act as independent evolutionary entities, and that the merger of these biosynthetic subunits is a mechanism that nature has used to generate structural novelty in secondary metabolism [3].

Among the MAR4 Orf2 PTases, only *nap* has been formally linked to its secondary metabolic products [14, 28]. HIBGC11 contains all of the genes required for phenazine biosynthesis (*phzA-G*), suggesting it may be responsible for lavanducyanin biosynthesis (Table 1). Surprisingly, however, the PTase in this cluster (PTC1) does not clade with the characterized sequences EpzP and PpzP, which are known to prenylate phenazine scaffolds [20]. HIBGC9 contains a type III polyketide synthase, suggesting it may encode the production of the marinone class of naphthoquinones. This HIBGC was observed in CNQ-509, which is known to produce compounds in the marinone series [9]. However, experimental evidence will be required to confirm this hypothesis. The strains containing HIBGC8 (CNB-632 and CNH-099) also produce marinones [9], suggesting this version of the gene cluster may be truncated due to inadequate sequencing (Table 1). It is difficult to predict the types of molecules produced by the remainder of the uncharacterized HIBGCs. There are examples where PTases are not clustered with the genes required to produce the entire HI scaffold [13, 36], creating additional challenges for structure prediction. Regardless, the large number of uncharacterized pathways identified in MAR4 strains suggests they possess the genetic potential to produce a greater diversity of HI secondary metabolites than formerly appreciated.

The *mev* pathway has previously been associated with HI biosynthesis [26]. From our analysis, only 13 of 120 strains contained this pathway, including 11 of 35 strains that contain an ABBA PTase (Additional file 1: Table S4). In the two strains that contain the *mev* pathway but lack an ABBA PTase, the *mev* pathway is associated with terpenoid biosynthetic genes suggesting that this isoprene is also incorporated into secondary metabolism. These observations lend support to the hypothesis that when present in *Streptomyces* genomes, the *mev* pathway provides isoprene for secondary metabolism. However, the infrequency of the *mev* pathway in ABBA PTase-containing genomes indicates that it is not a reliable marker for HI production.

## Conclusions

Overall, the results of this study support the hypothesis that MAR4 strains are enriched in the biosynthetic machinery required to produce HI secondary metabolite relative to other streptomycetes. We also show that gene duplication, HGT, and gene rearrangement are involved in the evolution of HIBGCs and, by extension, the generation of HI chemical novelty. Secondary metabolites have previously been linked to functional adaptation in actinobacteria [37], therefore the accumulation of HIs in this clade could be related to the colonization of a particular environmental niche. The goal of understanding why such a diversity of HI gene clusters have evolved and been maintained in MAR4 strains will require the challenging task of linking these molecules to their ecological roles in the environment.

## Methods

### Genome sequences

MAR4 strains were cultured in 200 mL of A1 medium (10 g soluble starch, 4 g yeast extract, 2 g peptone, 750 mL seawater, 250 mL deionized water) as previously described [4]. DNA was extracted according to the Joint Genome Institute (JGI) standard protocol (http://my.jgi.doe.gov/general/protocols.html). Genome sequencing, annotation, and assembly were carried out as previously described [4]. The genome sequence of MAR4 strain CNQ-509 was provided by Prof. Lutz Heide (University of Tuebingen, Germany) and will be made public as part of an upcoming publication [38]. An additional 108 publically available *Streptomyces* genome sequences were obtained from IMG (Additional file 1: Table S1; https://img.jgi.doe.gov/cgi-bin/er/main.cgi).

### Identification of ABBA prenyltransferase homologs

Seventeen characterized ABBA PTases of fungal and bacterial origin [20] were used as query sequences in a BLASTp search of the 120 *Streptomyces* genomes using the BLAST interface at IMG/ER (https://img.jgi.doe.gov/er/), with an e-value cutoff of 1e^−5^. The MAR4 strain CNQ-509, which was not available through JGI, was individually searched with the same query set using BLAST+ [39]. Multiple sequence alignments of all BLAST hits to characterized indole and Orf2 PTases were constructed independently using MUSCLE [40]. For each alignment, a maximum likelihood phylogeny was constructed using raxmlGUI [41] and the GTR + G model. For the Orf2 PTase phylogeny, two outgroups were included from the indole PTase family. Likewise, for the indole family, two Orf2 PTases were used as outgroups. A BLAST hit was identified as an Orf2 or indole ABBA PTase if it belonged to either of those clades and did not display an excessively long branch length.

### *Streptomyces* phylogeny

Five housekeeping genes (*recA, atpD, rpoB, gyrB*, and *trpB*) that have previously been used in *Streptomyces* multi-locus sequence typing [42] were identified in the set of 120 *Streptomyces* genomes using a BLAST search for each gene. The amino acid sequences were aligned using MUSCLE and individual maximum likelihood phylogenies built using raxmlGUI [41]. Gene identities were confirmed if they formed a monophyletic clade with their respective homologs. Housekeeping genes from two *Pseudonocardia* strains (*P. dioxanivorans* CB1190 and *P. asaccharolytica* DSM44247) were included as outgroups. Of the five housekeeping genes examined, only *rpoB* and *atpD* were present in single copy in all of the 120 genomes. These two housekeeping genes were thus selected to build a *Streptomyces* phylogeny. Multiple sequence alignments of these two genes were manually trimmed to the same length and concatenated. raxmlGUI was used to build a maximum likelihood phylogeny of the resulting multiple sequence alignment. LG + I + G was selected as the best-fit model for the data set based on a ProtTest [43] analysis of the concatenated alignment.

### Orf2 prenyltransferase phylogeny

An amino acid phylogeny of the Orf2 ABBA prenyltransferases was constructed using all homologs identified in the *Streptomyces* genome sequences and a set of characterized Orf2 prenyltransferases [20]. The sequences were aligned using MUSCLE [40] and a maximum likelihood phylogeny built using raxmlGUI [41] as describe above. Based on a ProtTest analysis [43], LG was chosen as the best-fit model for the data. A preliminary tree using two indole prenyltransferases as outgroups (SCO7190, accession number WP_011031680; CymD, accession number SARE_4565) was built to confirm that the Orf2 prenyltransferase sequences were monophyletic. To improve the alignment quality, the outgroups were removed, the sequences re-aligned, and a final tree built using the same methods with midpoint rooting.

### HI gene cluster identification

Gene clusters associated with all MAR4 PTases were analyzed using a combination of BLAST (http://blast.ncbi.nlm.nih.gov/Blast.cgi) and IMG/ER, (https://img.jgi.doe.gov/cgi-bin/er/main.cgi). Clusters were defined by shared content and gene annotation. Gene clusters in different strains were compared using MultiGeneBlast [34] and a database of the 12 MAR4 genome sequences. Gene clusters were considered to be the same if they shared >85 % gene content and had a MultiGeneBlast cumulative BLAST bit score >80 % of the query sequences’ score to itself. Cumulative BLAST bit scores dropped precipitously in strains that did not contain the cluster. Uncharacterized pathways were assigned “hybrid isoprenoid biosynthetic gene cluster” numbers (HIBGC 1-12). The previously characterized napyradiomycin (*nap*) pathway was assembled with the aid of the published sequence [14].

### MAR4 phylogeny

The five housekeeping genes used in a prior *Streptomyces* phylogeny [42] were all present in single copy in the 12 MAR4 genomes and used to build a more robust MAR4 species phylogeny. Nucleotide multiple sequence alignments for each of these genes and the 16S rRNA gene were individually built using MUSCLE [40] and manually trimmed to the same length. The six alignments were then concatenated and a maximum likelihood phylogeny built using raxmlGUI [41] with 100 bootstrap replicates using the GTR + G substitution model. A maximum parsimony tree was built using PAUP* [44] with 100 bootstrap replicates. The two strains that were the most closely related to the MAR4 clade in the *Streptomyces* phylogeny (*S. vitaminophilus* DSM 41686 and *S. bottropensis* ATCC 25435) were included as outgroups. Acquisition points of PTCs within the MAR4 lineage were predicted using the trace character history function in Mesquite [45] as previously described [4]. Likelihood scores >50 % were used to predict the points of PTC acquisition in the MAR4 phylogeny.

### Mevalonate pathway identification

To identify the mevalonate (*mev*) pathway, the 3-hydroxy-3-methyl-glutaryl-CoA reductase gene from *S. aculeolatus* NRRL 18422 (ABS50444) was used as a query in a BLASTp search of the *Streptomyces* genomes. This gene has previously been used as a marker for the *mev* pathway [29, 46]. Once located, the neighboring genes were manually examined to confirm the presence and synteny of the five remaining genes in the pathway (3-hydroxy-3-methyl-glutaryl-CoA synthase, isopentenyl pyrophosphate isomerase, phosphomevalonate kinase, mevalonate decarboxylase, and mevalonate kinase).

## Availability of supporting data

All genome sequences discussed in this study (with the exception of the sequence derived from strain CNQ-509) are available through the Joint Genome Institute Integrated Microbial Genomes database (https://img.jgi.doe.gov/cgi-bin/er/main.cgi) and are accessible through the identification numbers listed in Additional file 1: Table S1. The genome sequence of strain CNQ-509 will be made public as a part of an upcoming publication [38]. Primary phylogenetic data has been uploaded into TreeBase (http://purl.org/phylo/treebase/phylows/study/TB2:S18360).

## Additional file

Additional file 1:
**Table S1.** Genome sequences used in this study. **Table S2.** ABBA PTases used as query sequences. **Table S3.** List of ABBA PTases identified in this study. **Table S4.** List of strains containing the complete mevalonate pathway. (PDF 215 kb)

## Abbreviations

HI: Hybrid isoprenoid
PTase: Prenyltransferase
*mev*: Mevalonate biosynthetic pathway
HIBGC: Hybrid isoprenoid biosynthetic gene cluster
PTC: Prenyltransferase clade
*nap*: Napyradiomycin biosynthetic pathway
