# Supplementary material for: Genomic insights into the evolution of hybrid isoprenoid biosynthetic gene clusters in the MAR4 marine streptomycete clade
Source: BMC Genomics. 2015 Nov 17;16:960. doi: 10.1186/s12864-015-2110-3 (PMC4650096; doi:10.1186/s12864-015-2110-3)
Supplement: Additional file 1: — Table S1. Genome sequences used in this study. Table S2. ABBA PTases used as query sequences. Table S3. List of ABBA PTases identified in this study. Table S4. List of strains containing the complete mevalonate pathway. (PDF 215 kb) [file 12864_2015_2110_MOESM1_ESM.pdf]

Supplementary Tables

Table S1 Genome sequences used in this study. MAR4 genomes, with the exception of CNQ-509, were obtained as part of this study. The remaining streptomycete genomes represent those available through JGI as of March 2014.
*Sequence provided by Prof. Lutz Heide at the University of Tuebingen, Germany.
**Sequence no longer available from the JGI.

No.	Genome	IMG taxon ID	NCBI taxon ID
1	*Streptomyces* sp. CNB-632 (MAR4)	2558860984	1408314
2	*Streptomyces* sp. CNH-099 (MAR4)	2515154205	1137269
3	*Streptomyces* sp. CNP-082 (MAR4)	2526164782	1137270
4	*Streptomyces* sp. CNQ-525 (MAR4)	2561511112	418855
5	*Streptomyces* sp. CNQ-329 (MAR4)	2528311045	1298879
6	*Streptomyces* sp. CNQ-509 (MAR4)*	N/A	N/A
7	*Streptomyces* sp. CNQ-766 (MAR4)	2517572165	1169157
8	*Streptomyces* sp. CNQ-865 (MAR4)	2524023247	1288081
9	*Streptomyces* sp. CNS-335 (MAR4)	2517572166	1169160
10	*Streptomyces* sp. CNX-435 (MAR4)	2558860985	1408316
11	*Streptomyces* sp. CNY-243 (MAR4)	2518285564	1169161
12	*Streptomyces* sp. CNT-371 (MAR4)	2515154206	1136433
13	*Streptomyces acidiscabies* 84-104	2547132143	1116232
14	*Streptomyces afghaniensis* 772	2545824777	1283301
15	*Streptomyces albulus* CCRC 11814	2545824724	1316445
16	*Streptomyces albus* J1074	2541047081	457425
17	*Streptomyces aurantiacus* JA 4570	2545824776	1286094
18	*Streptomyces auratus* AGR0001	2531839540	1160718
19	*Streptomyces avermitilis* MA-4680	637000304	227882
20	*Streptomyces bingchenggensis* BCW-1	646862346	749414
21	*Streptomyces bottropensis* ATCC 25435	2517572239	1054862
22	*Streptomyces canus* 299MFChir4.1	2521172643	1172183
23	*Streptomyces cattleya* ATCC 35852	2504756050	29303
24	*Streptomyces chartreusis* NRRL 12338	2547132122	1079986
25	*Streptomyces chartreusis* NRRL 3882	2547132121	1079985
26	*Streptomyces clavuligerus* ATCC 27064	651324105	443255
27	*Streptomyces coelicoflavus* ZG0656	2534682028	1120227
28	*Streptomyces coelicolor* A3(2)	637000305	100226
29	*Streptomyces collinus* Tu 365	2554235367	1214242
30	*Streptomyces davawensis* JCM 4913	2561511188	1214101
31	*Streptomyces flavidovirens* DSM 40150	2522572192	1123319
32	*Streptomyces fulvissimus* DSM 40593	2554235391	1303692

33	*Streptomyces gancidicus* BKS 13-15	2537561976	1284664
34	*Streptomyces ghanaensis* ATCC 14672	645058824	566461
35	*Streptomyces globisporus* C-1027	2548877066	1172567
36	*Streptomyces griseoaurantiacus* M045	651324106	996637
37	*Streptomyces griseoflavus* Tu4000	645058728	467200
38	*Streptomyces griseus griseus* NBRC 13350	641522653	455632
39	*Streptomyces hygroscopicus* ATCC 53653	645058857	457427
40	*Streptomyces hygroscopicus jinggangensis* 5008	2561511169	1133850
41	*Streptomyces hygroscopicus jinggangensis* TL01	2561511180	1203460
42	*Streptomyces ipomoeae* 91-03	2537561795	698759
43	*Streptomyces lividans* TK24	645058856	457428
44	*Streptomyces lysosuperificus* ATCC 31396	2547132120	1079984
45	*Streptomyces mobaraensis* NBRC 13819	2537561865	1223523
46	*Streptomyces pristinaespiralis* ATCC 25486	648861016	457429
47	*Streptomyces purpureus* ATCC 21405	2516493006	1054860
48	*Streptomyces rimosus rimosus* ATCC 10970	2541047971	1265868
49	*Streptomyces roseosporus* NRRL 11379	645058827	457430
50	*Streptomyces roseosporus* NRRL 15998	645058822	457431
51	*Streptomyces scabiei* 87.22	646564576	680198
52	*Streptomyces scabrisporus* DSM 41855	2515154197	1123320
53	*Streptomyces somaliensis* DSM 40738	2548876818	1134445
54	*Streptomyces* sp. SirexAA-E	2523533511	862751
55	*Streptomyces* sp. 142MFCol3.1	2524614552	1172179
56	*Streptomyces* sp. 303MFCol5.2	2521172626	1172181
57	*Streptomyces* sp. 351MFTsu5.1	2521172628	1172180
58	*Streptomyces* sp. AA0539	2551306164	1210045
59	*Streptomyces* sp. AA1529	2551306127	1203257
60	*Streptomyces* sp. XyelbKG-1 1	647000328	649189
61	*Streptomyces* sp. Amel2xB2	2524614578	1305829
62	*Streptomyces* sp. Amel2xC10	2524614730	1305826
63	*Streptomyces* sp. C	645058853	253839
64	*Streptomyces* sp. CNB-091	2518285535	1169156
65	*Streptomyces* sp. CNH-189	2515154150	1136432
66	*Streptomyces* sp. CNH-287	2524023245	1288082
67	*Streptomyces* sp. CNR-698	2516143117	1206101
68	*Streptomyces* sp. CNS-606	2526164783	1305837
69	*Streptomyces* sp. CNS-615	2518285536	1169158
70	*Streptomyces* sp. CNS-654	2561511106	1931

71	*Streptomyces* sp. CNT-302	2517572190	1169155
72	*Streptomyces* sp. CNT-318	2524614559	1288079
73	*Streptomyces* sp. CNT-360	2524614560	1288080
74	*Streptomyces* sp. CNT-372	2517572167	1169154
75	*Streptomyces* sp. CNY-228	2517572184	1169159
76	*Streptomyces* sp. DpondAA-B6	2524614581	682311
77	*Streptomyces* sp. DvalAA-21	2524614543	1305824
78	*Streptomyces* sp. e14	647533235	645465
79	*Streptomyces* sp. FXJ7.023	2554235020	579932
80	*Streptomyces* sp. HGB0020	2541046998	1078086
81	*Streptomyces* sp. HPH0547	2541047017	1203592
82	*Streptomyces* sp. HrubLS-53	2504756065	60912
83	*Streptomyces* sp. KhCrAH-337	2524614544	1305839
84	*Streptomyces* sp. KhCrAH-40	2524614547	1305838
85	*Streptomyces* sp. KhCrAH-43	2524614576	1305827
86	*Streptomyces* sp. LaPpAH-201	2526164526	1305823
87	*Streptomyces* sp. LCC**	N/A	N/A
88	*Streptomyces* sp. Mg1	642791623	465541
89	*Streptomyces* sp. PAMC26508	2561511190	1265601
90	*Streptomyces* sp. PgraA7	2524614673	1157641
91	*Streptomyces* sp. PP-C42	2547132312	986330
92	*Streptomyces* sp. PsTaAH-130	2524614577	1305828
93	*Streptomyces* sp. PsTaAH-137	2524614580	1305830
94	*Streptomyces* sp. S4	2547132081	889487
95	*Streptomyces* sp. SA3_actG	649990018	683219
96	*Streptomyces* sp. ScaeMP-e122	2524614542	1305825
97	*Streptomyces* sp. SPB74	647533234	465543
98	*Streptomyces* sp. SPB78	645951849	591157
99	*Streptomyces* sp. SS	2551306143	260742
100	*Streptomyces* sp. TAA-040	2524614564	1288083
101	*Streptomyces* sp. TAA-204	2524614565	1289387
102	*Streptomyces* sp. TAA-486	2524614850	1298880
103	*Streptomyces* sp. TOR3209	2547132111	1073567
104	*Streptomyces* sp. Tu6071	651285011	355249
105	*Streptomyces* sp. W007	2514752031	1055352
106	*Streptomyces* sp. WMMB 322	2522125135	1286821
107	*Streptomyces* sp. WMMB 714	2522125136	1286822
108	*Streptomyces sulphureus* DSM 40104	2518645610	1123321
109	*Streptomyces sviceus* ATCC 29083	648861017	463191
110	*Streptomyces tsukubaensis* NRRL 18488	2529292926	1114943

111	*Streptomyces turgidiscabies* Car8	2541046981	698760
112	*Streptomyces venezuelae* ATCC 10712	2524023215	953739
113	*Streptomyces violaceusniger* SPC6	2554235005	1306406
114	*Streptomyces violaceusniger* Tu 4113	648276750	653045
115	*Streptomyces viridochromogenes* DSM 40736	645058855	591159
116	*Streptomyces viridochromogenes* Tue57	2531839509	1160705
117	*Streptomyces viridosporus* T7A, ATCC 39115	2518285526	665577
118	*Streptomyces vitaminophilus* DSM 41686	2515154142	1123322
119	*Streptomyces xinghaiensis* S187, NRRL B24674	2548876513	1038929
120	*Streptomyces zinciresistens* K42	2531839181	700597

Table S2 ABBA PTases used as query sequences (adapted from [1]).

Name [Reference]	NCBI Accession	Strain	Indole or Orf2	Associated product
PpzP [2]	CAX48655	*S. anulatus* 9663	Orf2	endophenazine
EpzP [3]	ADQ43372	*S. cinnamonensis* DSM 1042	Orf2	endophenazine
NapT9 [4]	ABS50462	*S. aculeolatus* NRRL 18422	Orf2	napyradiomycin
NapT8 [4]	ABS50461	*S. aculeolatus* NRRL 18422	Orf2	napyradiomycin
NphB [5]	1ZB6_A	*S.* sp. CL190	Orf2	naphterpin
Fnq26 [6]	CAL34104	*S. cinnamonensis* DSM 1042	Orf2	furanonaphthoquinone
Fur7 [7]	BAE78975	*S.* sp. KO-3988	Orf2	furaquinocin
CloQ [8]	AAN65239	*S. roseochromogenes* subsp. *oscitans* DS 12.976	Orf2	chlorobiocin
NovQ [9]	AAF67510	*S. niveus* DSM 40088	Orf2	novobiocin
Ptf_{At} [10]	EAU39467	*Aspergillus terreus* NIH2624	Orf2	unknown
Ptf_{Bf} [10]	EDN25735	*Botryotinia fuckeliana* B05.10	Orf2	unknown
Ptf_{Ss} [10]	EDN93598	*Sclerotinia sclerotiorum* 1980 UF-70	Orf2	unknown
LtxC [11]	AAT12285	*Lyngbia majuscula*	indole	lyngbiatoxin
CymD [12]	ABW00334	*Salinispora arenicola* CNS-205	indole	cyclomarin

IptA [13]	BAJ07990	*S.* sp. SN-593	indole	6-dimethylallylindole-3-carbaldehyde
7-DMATS [14]	ABS89001	*Aspergillus fumigatus*	indole	unknown
NotC [15]	ADM34131	*Aspergillus* sp. MF297-2	indole	notamide

Table S3 List of ABBA PTases identified in this study. MAR4 strains are in bold. N/A = not applicable.
*Refers to IMG gene identification numbers for all strains except *S.* sp. CNQ-509, for which gene numbers are those reported in [16].

Strain	Gene ID*	PTase	Closest query sequence (% AA identity)	PTC	HIBGC
S. acidiscabies 84-104	2547559206	indole	IptA (58%)	N/A	N/A
	2547553903	Orf2	PpzP (45%)	N/A	N/A
S. bottropensis ATCC 25435	2518245567	indole	IptA (59%)	N/A	N/A
S. clavuligerus ATCC 27064	651409133	indole	CymD (43%)	N/A	N/A
S. coelicoflavus ZG0656	2536362544	indole	IptA (63%)	N/A	N/A
S. coelicolor A3(2)	637271736 (SCO7467)	indole	IptA (59%)	N/A	N/A
	637271461 (SCO7190)	Orf2	EpzP (29%)	N/A	N/A
S. davawensis JCM 4913	2562412110	Orf2	Fur7 (78%)	N/A	N/A
S. gancidicus BKS 13-15	2539351921	Orf2	NphB (31%)	N/A	N/A
S. griseoflavus Tu4000	645416841	Orf2	PpzP (46%)	N/A	N/A
S. ipomoeae 91-03	2538658440	indole	IptA (60%)	N/A	N/A
S. lividans TK24	645400595	indole	IptA (59%)	N/A	N/A
	645400867	Orf2	EpzP (29%)	N/A	N/A
S. mobaraensis NBRC 13819	2538963887	Orf2	PpzP (44%)	N/A	N/A
S. rimosus rimosus ATCC 10970	2545388428	indole	IptA (31%)	N/A	N/A
S. scabiei 87.22	646659289	indole	IptA (56%)	N/A	N/A
S. scabrisporus DSM 41855	2516052848	indole	IptA (47%)	N/A	N/A
S. sp. 303MFCol5.2	2521676165	indole	LtxC (29%)	N/A	N/A
	2521683528	Orf2	Fnq26 (45%)	N/A	N/A
	2521683684	Orf2	NphB (52%)	N/A	N/A

S. sp. Amel2xC10	2525559524	indole	IptA (60%)	N/A	N/A
***S.* sp. CNB-632**	2561470561	Orf2	Fnq26 (43%)	PTC1	HIBGC11
	2561472124	Orf2	PpzP (48%)	PTC8	HIBGC5
	2561473002	Orf2	PpzP (40%)	PTC2	HIBGC4
	2561473004	Orf2	PpzP (38%)	PTC12	HIBGC4
	2561473755	Orf2	NphB (59%)	PTC4	HIBGC6
	2561476030	Orf2	NapT9 (48%)	PTC7	HIBGC8
	2561476124	Orf2	NapT9 (44%)	PTC13	HIBGC2
	2561476125	Orf2	Fnq26 (51%)	PTC11	HIBGC5
***S.* sp. CNH-099**	2516097927	Orf2	Fnq26 (43%)	PTC1	HIBGC11
	2516099186	Orf2	PpzP (48%)	PTC13	HIBGC2
	2516101115	Orf2	NphB (59%)	PTC4	HIBGC6
	2516101746	Orf2	PpzP (38%)	PTC12	HIBGC4
	2516101748	Orf2	PpzP (40%)	PTC2	HIBGC4
	2516104298	Orf2	NapT9 (48%)	PTC7	HIBGC8
	2516104521	Orf2	Fnq26 (52%)	PTC11	HIBGC5
	2516104522	Orf2	NapT9 (43%)	PTC8	HIBGC5
S. sp. CNH-189	2515835839	Orf2	NapT9 (45%)	N/A	N/A
S. sp. CNH-287	2524586714	Orf2	NphB (51%)	N/A	N/A
	2524588539	Orf2	PpzP (55%)	N/A	N/A
***S.* sp. CNP-082**	2527270384	Orf2	NphB (42%)	PTC3	HIBGC7
	2527270866	Orf2	NapT8 (93%)	PTC10	*nap*
	2527270867	Orf2	NapT9 (80%)	PTC6	*nap*
***S.* sp. CNQ-525**	2562118558	Orf2	PpzP (42%)	PTC1	HIBGC11
	2562121671	Orf2	PpzP (49%)	PTC13	HIBGC2
	2562123443	Orf2	NphB (42%)	PTC9	HIBGC1
	2562123520	Orf2	NapT8 (100%)	PTC10	*nap*
	2562123521	Orf2	NapT9 (99%)	PTC6	*nap*
***S.* sp. CNQ-509**	AA958_07645	indole	IptA (29%)	N/A	HIBGC10
	AA958_30735	Orf2	Fnq26 (41%)	PTC1	HIBGC11
	AA958_24325	Orf2	NphB (65%)	PTC5	HIBGC9
	AA958_24270	Orf2	NapT9 (47%)	PTC7	HIBGC9
	AA958_18620	Orf2	NphB (62%)	PTC4	HIBGC6
	AA958_12645	Orf2	PpzP (38%)	PTC2	HIBGC3
	AA958_12635	Orf2	Fnq26 (53%)	PTC11	HIBGC3
	AA958_12625	Orf2	NapT9 (44%)	PTC8	HIBGC3
***S.* sp. CNQ-329**	2528489393	Orf2	NapT9 (41%)	PTC9	HIBGC1
	2528491298	Orf2	NphB (42%)	PTC3	HIBGC7
	2528491789	Orf2	NapT8 (93%)	PTC10	*nap*
	2528491790	Orf2	NapT9 (80%)	PTC6	*nap*
***S.* sp. CNQ-766**	2518002741	Orf2	PpzP (42%)	PTC1	HIBGC11
	2518005124	Orf2	PpzP (48%)	PTC13	HIBGC2
	2518006200	Orf2	NapT9 (41%)	PTC9	HIBGC1
	2518006639	Orf2	NapT9 (100%)	PTC6	*nap*
	2518006640	Orf2	NapT8 (100%)	PTC10	*nap*
***S.* sp. CNQ-865**	2524593636	Orf2	PpzP (49%)	PTC13	HIBGC2
	2524596589	Orf2	PpzP (42%)	PTC1	HIBGC11
	2524600624	Orf2	NphB 42%)	PTC9	HIBGC1
	2524600745	Orf2	NapT8 (100%)	PTC10	*nap*
	2524600746	Orf2	NapT9 (100%)	PTC6	*nap*

***S.* sp. CNS-335**	2518010821	Orf2	PpzP (49%)	PTC13	HIBGC2
	2518010999	Orf2	PpzP (42%)	PTC1	HIBGC11
	2518013585	Orf2	NphB (42%)	PTC9	HIBGC1
	2518013945	Orf2	NapT8 (100%)	PTC10	*nap*
	2518014051	Orf2	NapT9 (100%)	PTC6	*nap*
S. sp. CNS-654	2562084974	Orf2	PpzP (91%)	N/A	N/A
***S.* sp. CNT-371**	2516104881	Orf2	PpzP (48%)	PTC13	HIBGC2
	2516108106	Orf2	PpzP (42%)	PTC1	HIBGC11
	2516111586	Orf2	NphB 41%)	PTC9	HIBGC1
	2516112034	Orf2	NapT8 (98%)	PTC10	*nap*
	2516112035	Orf2	NapT9 (79%)	PTC6	*nap*
***S.* sp. CNX-435**	2561476450	Orf2	PpzP (48%)	PTC13	HIBGC2
	2561478010	Orf2	Fnq26 (42%)	PTC1	HIBGC11
	2561479929	Orf2	NphB (60%)	PTC4	HIBGC6
	2561481643	Orf2	PpzP (41%)	PTC2	HIBGC12
***S.* sp. CNY-243**	2518513180	Orf2	PpzP (42%)	PTC1	HIBGC11
	2518518536	Orf2	PpzP (49%)	PTC13	HIBGC2
	2518518703	Orf2	NapT9 (41%)	PTC9	HIBGC1
	2518518936	Orf2	NapT9 (100%)	PTC6	*nap*
	2518518937	Orf2	NapT8 (100%)	PTC10	*nap*
S. sp. FXJ7.023	2554329132	indole	IptA (63%)	N/A	N/A
S. sp. TOR3209	2547410785	indole	IptA (44%)	N/A	N/A
	2547405602	indole	IptA (56%)	N/A	N/A
S. violaceusniger Tu 4113	648755156	indole	IptA (64%)	N/A	N/A
S. vitaminophilus DSM 41686	2515785691	Orf2	Fnq26 (57%)	N/A	N/A

Table S4 Strains containing the complete mevalonate pathway, the location of the pathway in each genome, and the distance to the nearest ABBA PTase. N/A = not applicable (no ABBA PTase present).

Strain	Location of mevalonate pathway (JGI gene IDs)	Proximity to ABBA PTases
Streptomyces davawensis JCM 4913	2562412117-2562412112	1303 bp (1 ORF)
Streptomyces griseoflavus Tu4000	645416842-645416847	69 bp (0 ORFs)
Streptomyces sp. 303MFCol5.2	2521683690-2521683695	7,931 bp (5 ORFs)
Streptomyces sp. CNH-189	2515835853-2515835858	18,320 bp (13 ORFs)
Streptomyces sp. CNQ-865 (MAR4)	2524600726-2524600731	16,532 (13 ORFs)
Streptomyces sp. CNP-082 (MAR4)	2527270597-2527270602	Unknown - short contig
Streptomyces sp. CNQ-766 (MAR4)	2518006507-2518006512	Unknown - short contig
Streptomyces sp. CNS-335 (MAR4)	2518013953-2518013958	Unknown - short contig
Streptomyces sp. CNT-371 (MAR4)	2516111891-2516111896	Unknown - short contig
Streptomyces sp. CNQ-525 (MAR4)	2562123501-2562123506	16,498 bp (13 ORFs)
Streptomyces sp. CNY-243 (MAR4)	2518518916-2518518922	Unknown - short contig
Streptomyces sp. TAA-040	2524957321-2524957326	N/A
Streptomyces sp. CNT-372	2518017234-2518017240	N/A

References

1. Bonitz T, Alva V, Saleh O, Lupas AN, Heide L. Evolutionary relationships of microbial aromatic prenyltransferases. PLoS One. 2011;6:e27336.
2. Saleh O, Gust B, Boll B, Fiedler H-P, Heide L. Aromatic prenylation in phenazine biosynthesis: dihydrophenazine-1-carboxylate dimethylallyltransferase from *Streptomyces anulatus*. J Biol Chem. 2009;284:14439–47.
3. Seeger K, Flinspach K, Haug-Schifferdecker E, Kulik A, Gust B, Fiedler H-P, Heide L. The biosynthetic genes for prenylated phenazines are located at two different chromosomal loci of *Streptomyces cinnamonensis* DSM 1042. Microb Biotechnol. 2011;4:252–62.
4. Winter JM, Moffitt MC, Zazopoulos E, McAlpine JB, Dorrestein PC, Moore BS. Molecular basis for chloronium-mediated meroterpene cyclization - Cloning, sequencing, and heterologous expression of the napyradiomycin biosynthetic gene cluster. J Biol Chem. 2007;282:16362–8.
5. Kuzuyama T, Noel JP, Richard SB. Structural basis for the promiscuous biosynthetic prenylation of aromatic natural products. Nature. 2005;435:983–7.
6. Haagen Y, Glueck K, Fay K, Kammerer B, Gust B, Heide L. A gene cluster for prenylated naphthoquinone and prenylated phenazine biosynthesis in *Streptomyces cinnamonensis* DSM 1042. Chembiochem. 2006;7:2016-27.
7. Kumano T, Tomita T, Nishiyama M, Kuzuyama T. Functional characterization of the promiscuous prenyltransferase responsible for furaquinocin biosynthesis: identification of a physiological polyketide substrate and its prenylated reaction products. J Biol Chem. 2010;285:39663–71.
8. Pojer F, Wemakor E, Kammerer B, Chen H, Walsh CT, Li S-M, Heide L. CloQ, a prenyltransferase involved in clorobiocin biosynthesis. Proc Natl Acad Sci USA. 2003;100:2316–21.
9. Ozaki T, Mishima S, Nishiyama M, Kuzuyama T. NovQ is a prenyltransferase capable of catalyzing the addition of a dimethylallyl group to both phenylpropanoids and flavonoids. J Antibiot (Tokyo). 2009;62:385–92.
10. Haug-Schifferdecker E, Arican D, Brückner R, Heide L. A new group of aromatic prenyltransferases in fungi, catalyzing a 2,7-dihydroxynaphthalene 3-dimethylallyl-transferase reaction. J Biol Chem. 2010;285:16487–94.
11. Edwards DJ, Gerwick WH. Lyngbyatoxin biosynthesis: Sequence of biosynthetic gene cluster and identification of a novel aromatic prenyltransferase. J Am Chem Soc. 2004;126:11432–3.
12. Schultz AW, Oh DC, Carney JR, Williamson RT, Udwary DW, Jensen PR, Gould SJ, Fenical W, Moore BS. Biosynthesis and structures of cyclomarins and cyclomarazines, prenylated cyclic peptides of marine actinobacterial origin. J Am Chem Soc. 2008;130:4507–16.

13. Takahashi S, Takagi H, Toyoda A, Uramoto M, Nogawa T, Ueki M, Sakaki Y, Osada H. Biochemical Characterization of a novel indole prenyltransferase from *Streptomyces* sp SN-593. J Bacteriol. 2010;192:2839-51.
14. Kremer A, Westrich L, Li S-M. A 7-dimethylallyltryptophan synthase from *Aspergillus fumigatus*: overproduction, purification and biochemical characterization. Microbiology. 2007;153:3409–16.
15. Ding Y, de Wet JR, Cavalcoli J, Li S, Greshock TJ, Miller KA, Finefield JM, Sunderhaus JD, McAfoos TJ, Tsukamoto S, Williams RM, Sherman DH. Genome-based characterization of two prenylation steps in the assembly of the stephacidin and notoamide anticancer agents in a marine-derived *Aspergillus* sp. J Am Chem Soc. 2010;132:12733–40.
16. Rueckert C, Leipoldt F, Zeyhle P, Fenical W, Jensen PR, Kalinowski J, Heide L, Kaysser L. Complete genome sequence of *Streptomyces* sp. CNQ-509, a prolific producer of meroterpenoid chemistry. Submitted.
